# Carbon-Halloysite Nanocomposites and Their Adsorption Characteristics for Pharmaceuticals—A Naproxen Case Study

**DOI:** 10.3390/ma18112433

**Published:** 2025-05-22

**Authors:** Piotr Słomkiewicz, Beata Szczepanik, Piotr Sakiewicz, Klaudiusz Gołombek, Krzysztof Piotrowski

**Affiliations:** 1 Institute of Chemistry, Jan Kochanowski University, 25-406 Kielce, Poland; piotr.slomkiewicz@ujk.edu.pl (P.S.); beata.szczepanik@ujk.edu.pl (B.S.); 2 Department of Engineering Materials and Biomaterials, Faculty of Mechanical Engineering, Silesian University of Technology, 44-100 Gliwice, Poland; piotr.sakiewicz@polsl.pl; 3 Materials Research Laboratory, Faculty of Mechanical Engineering, Silesian University of Technology, 44-100 Gliwice, Poland; 4 Department of Chemical Engineering and Process Design, Faculty of Chemistry, Silesian University of Technology, 44-100 Gliwice, Poland

**Keywords:** halloysite, carbon, composite, naproxen, pharmaceuticals, adsorption

## Abstract

The synthesis of carbon-halloysite nanocomposites was carried out using aqueous sucrose solutions as a carbon precursor. Raw and calcined halloysite with different grain size classes were used as a carbon support. The influence of halloysite grain size and the calcination process on the carbon concentration in the composites and their adsorption characteristics towards the separation of naproxen from aqueous solutions was identified experimentally. The kinetic conditions of the process (pseudo-second-order kinetic model) indicate a favorable increase in the number of active sites formed after the deposition of the carbon layer on the surface of halloysite particles. Validation of the Langmuir multi-center isotherm adsorption model indicates a separation mechanism associated with the occurrence of multiple active centers on the nanocomposite adsorbent surface and the effect of separation without dissociation of naproxen particles. The obtained carbon-halloysite nanocomposite, due to the relatively cheap and simple, environmentally friendly production methodology and the required inexpensive raw materials, can be widely used in effective and common, economical treatment of wastewater streams from naproxen. The observed naproxen separation process effects are significant.

## 1. Introduction

One of the most commonly used pharmaceuticals in the world is naproxen (NPX)—a bicyclic propionic acid derivative, with complex but effective pharmaceutical effect [1,2,3,4,5,6,7]. It represents a pharmaceutical product classified as non-selective, non-steroidal anti-inflammatory drugs. Reaching natural ecosystems, naproxen and its metabolites (mainly O-desmethylnaproxen and naproxen glucuronide) show toxic effects on the organisms (bioaccumulation, introduction to the food chain in the ecosystem, modification of immune system and growth characteristics, feminization, hermaphroditism in fish population, mutagenic alteration in bacterial population, affecting the homeostasis and endocrine systems [7,8,9,10,11,12,13,14,15,16,17,18,19,20,21]).

Naproxen, due to its physicochemical properties [22], introduced into the natural environment is subject to various simultaneous physical, chemical and biological interactions [1,23]. In particular, the effects of sorption processes are directly dependent on the pH of the environment. This results from the chemical structure of naproxen, in which the carboxylic acid group occurs. The carboxyl group in the pH range of 5–8 (usually observed in the natural hydrosphere) is deprotonated, so that naproxen in the environment most often occurs in the anion form [22,23]. At the same time, the anion form significantly hinders or weakens the potential electrostatic interactions of naproxen with negatively charged natural clay mineral surfaces or with various types of natural organic substances in the environment [23].

In the surface waters, naproxen is exposed to natural solar radiation and undergoes direct/indirect photochemical degradation with the participation of carbonate, ferrous, ferric, nitrate ions, humic acids, pharmaceutical pollutants representing complex biologically active organic molecules and also producing reactive oxygen species (singlet oxygen, superoxide ions, hydroxyl radicals) [1,5,22,24,25,26,27,28]. Secondary pollutants (such as oxidation by-products, ferric sludge, ferrous oxide sludge, radical scavengers) can also be generated during improperly designed separation processes using chemical reactions (such as advanced oxidation processes including the Fenton approach or hybrid strategies like photo-Fenton, sono-Fenton, photochemical or various photo-catalytic processes, sonophotolysis, photoelectrocatalysis, advanced electrochemical oxidation [29], photocatalytic degradation [30], ozonation, photocatalytic ozonation or sonochemical approach) [2].

Naproxen is also resistant to potential microbial degradation bioprocesses because of its relatively high stability (the presence of two condensed rings) [1]. Various variants of complex (enzymatic) biotransformation of naproxen involving bacteria, fungi and algae are reported, with emphasis on relatively low process efficiency [11,31,32,33,34,35,36,37,38,39,40]. Moreover, biological neutralization of NPX is expensive and long [2]. A possibly cheap, effective method of NPX separation from aqueous solutions is sought, not based on chemical reactions and uncontrolled synthesis of reaction by-products (secondary contaminants), with a potentially even greater degree of risk to the natural environment than naproxen itself [22]. Current directions of research should be focused on physical processes. However, filtration and reverse osmosis processes are associated with high energy demand [2].

Considering these limitations, one of the methods of physical separation of NPX from aqueous solutions may be adsorption. Adsorption processes have many advantages, including reliability, mild exploitation conditions, high separation efficiency (over 90%), minimal energy demand of the process, as well as no simultaneous generation of secondary pollutants and the possibility of adsorbent regeneration [2]. However, the key problem is to select or even design, develop and produce in non-laboratory conditions (e.g., industrial) an appropriate adsorbent, both in terms of its unique tailored surface properties (selectivity), as well as with an appropriate internal structure (pore size distribution affecting the rate of internal diffusional phenomena) [2]. Potentially useful adsorbents in the separation of naproxen from aqueous solutions may be activated carbon [41,42], granular AC Bituminous coal (F400) [43], olive waste cake AC [44], metal–organic frameworks [45], silica-based porous materials (HMS) [42], chitosan-modified waste tire crumb rubber [46], green copper nano-adsorbent [47], activated biochar (N-biochar) [48], activated biochar (O-biochar) [48], and carbon-based magnetic adsorbent [49,50].

In the literature, there are descriptions of more complex adsorption systems for NPX removal, such as Fe_3_O_4_@SiO_2_/TMCc/GPTMS [51], based on graphene (GA) [50], silica-magnetic/graphene oxide [52], Al-MOF-Fe_3_O_4_@P4VP [53] or technically advanced prototype molecularly imprinted polymers (MIPs) (based on engineering of specific recognition sites in synthetic polymers [2]). Due to the technological complexity of the production processes, GA-based adsorbents and MIPs are not used on a mass scale in wastewater treatment.

Nanocomposites based on simple, possibly inexpensive substrates and relatively simple methods of their preparation may become a market-demanded alternative, especially for molecularly imprinted polymers. Their widespread use should, however, be directly related to the possibility of technically tailoring their composition and structure, which is usually strongly correlated with their separation abilities.

The aim of the presented work was to experimentally verify the possibility of adsorptive removal of naproxen from aqueous solutions using a nanocomposite adsorbent represented by a layered aluminosilicate—halloysite with a deposited carbon. A relatively simple method of obtaining this type of nanocomposite, its separation capabilities of naproxen from aqueous solutions and available and inexpensive components necessary for its production can ensure its widespread use in water purification technologies.

## 2. Experimental

### 2.1. Materials and Reagents

The following materials were used in the study: raw halloysite from the Dunino mine (HS) (Intermark, Gliwice, Poland), sucrose (Diamant, Środa Wielkopolska, Poland), naproxen ((S)-(+)-2-(6-methyl-2) propionic acid) of 99% purity (Alfa Aesar, Karlsruhe, Germany), analytically pure methanol (ChemLand, Stargard, Poland), and deionized water (0.06 µS cm^−1^, measured at 24 °C). Table 1 summarizes selected properties of naproxen.

Halloysite from the Dunino deposit is a weathering product of basaltic rocks. The Dunino mine (Poland) is an open pit mine. The raw material has a homogeneous composition throughout the cross-section, there are no vein-type intergrowths and inclusions. The mineral composition of the geological mineral is as follows [54,55,56]:

- halloysite: 75.80%,
- iron oxides (mainly hematite and magnetite): 18.22%,
- iron and titanium oxides (mainly ilmenite): 2.4%.

The chemical formula of hydrated halloysite is: Al_2_Si_2_O_5_(OH)_4_ × *n*H_2_O. The water is interstitial water. In nature, there are two types of this mineral, differing in the number of water molecules: tetrahydrate (*n* = 4, termed halloysite 10 Å) and dihydrate (*n* = 2, termed halloysite 7 Å). Raw and dried halloysite have a similar structure, and differ only in the water content within the structure. Inter-layer water is removed from dried halloysite. This is the so-called dehydration process, i.e., the evaporation of hygroscopic water adsorbed on mineral grains and removal of fine water contained between the layers of clay minerals. Further heating of the mineral involves roasting above the temperature of 550–600 °C, which causes its dehydroxylation and transformation into a new structural form. Dehydroxylation of halloysite, as well as other clay minerals, causes some characteristic change in their structure. The dehydroxylation reaction proceeds according to stoichiometry Equation (1):

Al_2_Si_2_O_5_(OH)_4_ → Al_2_Si_2_O_7_ + 2H_2_O (1)

When heated at temperature values above 500 °C halloysite releases water from the crystal structure of the mineral and forms metahalloysite. Complete removal of water molecules from the halloysite structure requires high temperature values (about 450–500 °C). Heating halloysite causes a number of significant changes in its crystal structure. Dehydroxylation of halloysite occurs from about 500 °C up to about 900 °C. Beyond 1200 °C mullite is formed. Structural and morphological changes in halloysite closely accompany the heating process sequences. Initially, the structure of halloysite does not change because it is thermally stable. Up to about 900 °C its morphology and porosity do not change. Calcination at higher temperature levels causes disintegration (distortion) of channels in the original structure of the mineral, and thus reduces its porosity. Another important modification in the structure of halloysite during the calcination process is the formation of hydroxyl groups on the outer surface of halloysite nanotubes (which occurs at about 600–900 °C).

### 2.2. Preparation of Carbon-Halloysite Nanocomposites

The raw halloysite (HS) was dried, ground and washed with demineralized water. The 0.250–0.125 mm size fraction of raw halloysite was used.

For the purposes of the study, calcined halloysite (HK) was also prepared, obtained by the calcination process—heating halloysite below its melting point to cause partial chemical decomposition of this compound. In this case, calcination caused the removal of water from its crystal lattice together with some chemical surface properties modification (formation of hydroxyl groups).

The grain fractions used in the studies were: 1.000–0.500 mm, 0.500–0.250 mm, and 0.250–0.125 mm.

The carbon-halloysite nanocomposites were obtained by impregnating the appropriate halloysite fraction with an aqueous solution of sucrose, of a specified concentration (15, 30 and 45 wt.% of sucrose) and then carbonization was carried out under strictly defined conditions. A 20.0 g halloysite sample was added into 100 cm^3^ of an aqueous solution of sucrose of a specified concentration, and placed in an ultrasonic bath for 1 h to remove air bubbles. Then, the mixture was shaken using a shaker at a rate of 130 rpm for 24 h at temperature 25 °C. After filtering the sucrose solution, the obtained material was dried in a laboratory oven at 100 °C for 24 h. The dry material was placed in a quartz vessel and then carbonized in a Nabertherm HTC 08/16 furnace under a nitrogen atmosphere. During carbonization, the temperature was increased with 5 °C min^−1^ rate in a nitrogen atmosphere up to 400 °C, this temperature value was maintained for 2 h, then the temperature was again raised up to 800 °C and the material was left under such conditions for 8 h. The obtained carbon-halloysite material was ground in a Mono PULVERISETTE 6 ball mill and then sieved to obtain fractions with grain sizes from 0.05 up to 0.20 mm.

The obtained carbon-halloysite nanocomposite materials were designated for further study as (wt.%—of sucrose in aqueous impregnating solution):

(1) Raw halloysite: 15HS (15 wt.%—HS), 30HS (30 wt.%—HS), 45HS (45 wt.%—HS),
(2) Calcined halloysite: 15HK1 (15 wt.%—HK_1.00–0.500_), 30HK1 (30 wt.%—HK_1.00–0.500_), 45HK1 (45 wt.%—HK_1.00–0.500_),
(3) Calcined halloysite: 15HK05 (15 wt.%—HK_0.500–0.250_), 30HK05 (30 wt.%—HK_0.500–0.250_), 45HK05 (45 wt.%—HK_0.500–0.250_),
(4) Calcined halloysite: 15HK025 (15 wt.%—HK_0.250–0.125_), 30HK025 (30 wt.%—HK_0.250–0.125_), 45HK025 (45 wt.%—HK_0.250–0.125_).

### 2.3. Characteristics of Carbon-Halloysite Nanocomposites

Morphological and textural properties of the carbon-halloysite nanocomposites were identified using the methods of low-temperature nitrogen adsorption–desorption isotherms on a volumetric adsorption analyzer ASAP 2020 by Micromeritics (Norcross, GA, USA) (Structural Research Laboratory of Jan Kochanowski University in Kielce, Poland). Before measurement tests, all of the samples were degassed at a temperature 200 °C for 2 h. Specific surface area (*S*_BET_) of explored carbon-deposited materials was determined with the use of Brunauer–Emmett–Teller (BET) interpretation method. The relative pressure range from 0.05 to 0.2 was applied, using the surface occupied by a single molecule of nitrogen in an adsorptive monolayer (assumed cross-sectional area 0.162 nm^2^ [57,58]). Total pore volume (*V*_t_) (interpreted as the sum of micropore volume (*V*_mi_) and volume of mesopores (*V*_me_)) was then calculated from one point of nitrogen adsorption isotherm, corresponding to the relative pressure *p*/*p*_o_ = 0.99 [58].

The SEM images of the prepared material samples were obtained by scanning electron microscopy–energy-dispersive X-ray spectroscopy (SEM–EDS) measurements carried out on Zeiss Supra 35 at an accelerating voltage of 15 kV and magnifications of 200–5000× in Silesian University of Technology, Gliwice, Poland. Secondary electron detector SE and backscatter electron detector BSE were used for the study. Chemical composition analyses were performed using Thermo Scientific™ EDX UltraDry X-ray energy detector with Pathfinder software (Pathfinder X-Ray Microanalysis Software ver. 2.4).

Infrared spectra were measured using a PerkinElmer Spectrum 400 FT-IR/FT-NIR spectrometer with a smart endurance single bounce diamond, attenuated total reflection (ATR) cell. Spectra in the 4000–650 cm^−1^ range were measured by the co-addition of 40 scans with a resolution of 4 cm^−1^.

The analysis of the particle size distribution for adsorbents was performed using a Fritsch device, model Analysette 22 MicroTec plus, which uses the phenomenon of laser diffraction for measurement. In this device, the source of electromagnetic radiation are two semiconductor lasers with wavelengths λ = 532 nm and λ = 940 nm. This professional device allows for determining the particle size distribution in the measuring range from 0.08 to 2000 μm.

### 2.4. Adsorption Measurements

**Figure 1:**
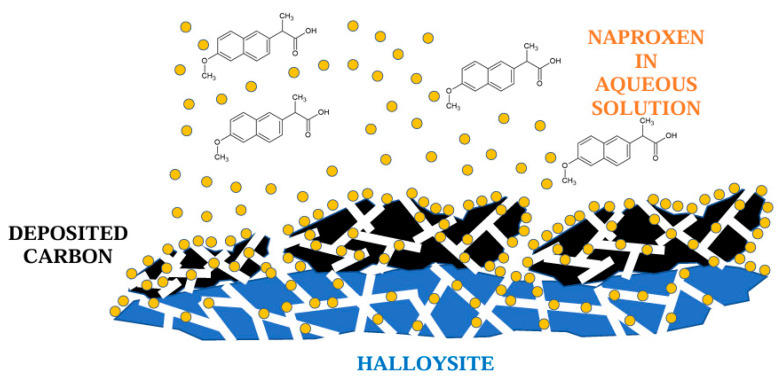
Carbon-halloysite nanocomposite and its use in the separation of naproxen from the liquid phase.

Batch adsorption experiments were performed in a 100 cm^3^ Erlenmeyer’s flask containing carbon-halloysite nanocomposite and the adsorbate aqueous solution, which were put into the incubator for 5, 10, 15, 30, 45, 60, 90, 120, 180, 240, 300, 360 and 1440 min. The temperature during measurement was 25 °C and the mixing rate was set to 120 rpm. Concentration values of adsorbate solutions were determined with the spectrophotometric method (UV Shimadzu UV-1800 spectrophotometer). The wavelength used to determine solution concentrations of naproxen was 231 nm. The removal efficiency (*R*, %) and the adsorption capacity (*q_e_*, mg g^−1^) were calculated using Equations (2) and (3):

(2) $$R\;[\%]=(\frac{C_{o}-C_{e}}{C_{o}}\;)\times 100\%$$

(3) $$q_{e}=\frac{(\;C_{o}-C_{e\;})\times V}{m}\;$$

where *C*_0_, *C_e_* (mg dm^−3^)—initial and equilibrium concentration values of adsorbate solutions, *V* (dm^3^)—the volume of the adsorbate solution, and *m* (g)—the mass of adsorbent. The separation of naproxen from the liquid phase using the investigated carbon-halloysite nanocomposite is schematically presented in Figure 1.

## 3. Results and Discussion

### 3.1. Characteristics of Adsorbents (Nanocomposites)

Structural parameters of the obtained carbon-halloysite nanocomposites were calculated from the adsorption–desorption N_2_ isotherms. The values of Brunauer–Emmett–Teller (BET) specific surface area (*S*_BET_), total pore volume (*V*_t_), volume of mesopores (*V*_me_), micropores (*V*_mi_), and mesoporosity fraction (%) are presented in Table 2. The *S*_BET_ decreased for all nanocomposites in comparison with unmodified halloysite (HS) or calcined halloysite (HK) except for the 45HS nanocomposite, for which an increase in this value is observed. Carbon deposition on the halloysite surface caused some decrease in the total pore volume and volume of mesopores, while the volume of micropores increased for nanocomposite samples, especially for 45HS and 45HK samples in comparison to the raw and calcined halloysite support.

Analyzing the data from Table 2, a significant decrease in the available adsorption surface of halloysite, both raw (HS) and calcined (HK), can be seen. The greatest decrease in the *S*_BET_ surface value is visible for the lowest tested sucrose concentration in the impregnating solution (for HS it is 45.64 → 27.23 m^2^∙g^−1^, for HK these are: 62.58 → 29.67 m^2^∙g^−1^, 69.74 → 33.73 m^2^∙g^−1^, 64.01 → 30.35 m^2^∙g^−1^ changes, respectively). These changes may result from the replacement (i.e., unavailability) of a certain fraction of the halloysite surface caused by the deposition of a carbon layer with a different surface morphology, which caused a clear change in the available adsorption geometry. This is also evidenced by the parallel change in the value of the *V*_t_ parameter. As a result of the deposition of a carbon layer on the surface, especially on the inner surface of halloysite support, the cross-sectional area of the capillaries (and therefore the diameter) is reduced. In the case of HS, these changes amounted to 0.1925 → 0.1348 cm^3^∙g^−1^, and for HK, these were: 0.1896 → 0.1423 cm^3^∙g^−1^, 0.1891 → 0.1611 cm^3^∙g^−1^, and 0.1757 → 0.1410 cm^3^∙g^−1^, respectively.

During the preparation process of carbon-halloysite nanocomposite systems, a carbon layer was formed on the external and internal surfaces of the halloysite carrier. This was associated first with the adsorption of sucrose from the aqueous solution inside the original internal pore structure of the halloysite carrier, and then with the process of the formation of a carbon layer at appropriately high temperature conditions. The produced carbon layer, with a specific volume, caused a significant reduction in the pore diameters of the original halloysite structure (and thus an observed reduction in the mesopores fraction).

At the same time, the characteristic porous structure of the carbon layer itself contributed to the appearance of additional micropores within the overall structure of the nanocomposite. As a result, a shift in the decisive pore volume fraction from the mesopore range towards micropores was observed.

**Figure 2:**
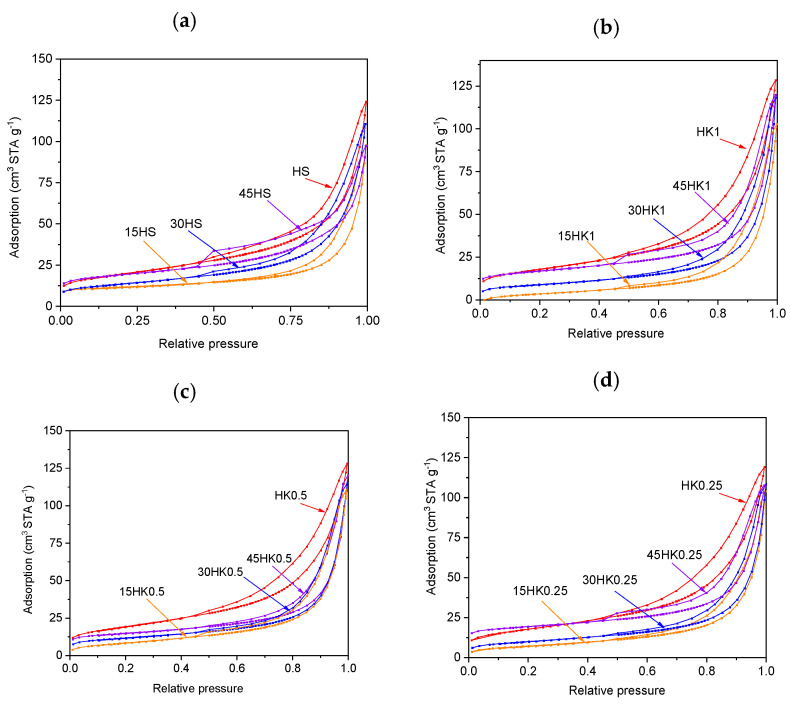
The nitrogen adsorption–desorption isotherms for HS, 15HS, 30HS, 45HS samples (**a**); HK1, 15HK1, 30HK1, 45HK1 samples (**b**); HK05, 15HK05, 30HK05, 45HK05 samples (**c**); HK025, 15HK025, 30HK025, 45HK025 samples (**d**).

Experimental adsorption–desorption N_2_ isotherms of the considered samples (Figure 2) are type IV according to the IUPAC classification [59], which, according to theoretical interpretation, indicates mesoporous characteristic of these solid materials. Numerical values of structural parameters derived from interpretation of adsorption isotherms are shown in Table 2.

However, increasing the concentration of the precursor (sucrose) in the solution causes a clear, systematic increase (reconstruction) of the available adsorption surface. This trend is observed for both raw halloysite (HS) and calcined halloysite (HK). In the case of HS, using 45% by weight of the sucrose aqueous solution, even the specific surface area *S*_BET_ of 54.75 m^2^∙g^−1^ can be obtained, exceeding by about 20% the value of this material parameter in relation to the halloysite carrier itself (45.64 m^2^∙g^−1^). In the case of different size fractions of physically modified halloysite (calcination, HK), the *S*_BET_ values obtained were: 67%, 60% and 66% of the reference HK value (without a deposited carbon layer). However, the change in the available *S*_BET_ value corresponds to a significant modification of the surface properties of the nanocomposite (mainly functional groups and surface morphology).

**Figure 3:**
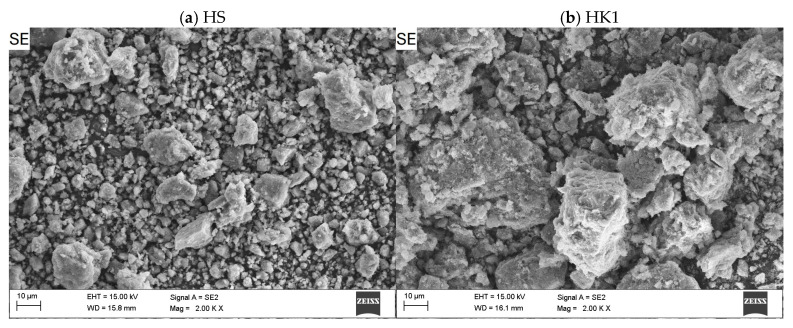

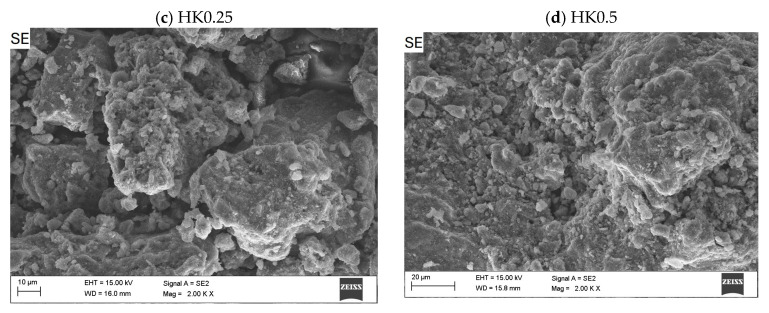
Surface morphology of raw halloysite (HS); calcined halloysite HK1, HK0.25, and HK0.5 (SEM, detector SE, magnification 2000×).

**Figure 4:**
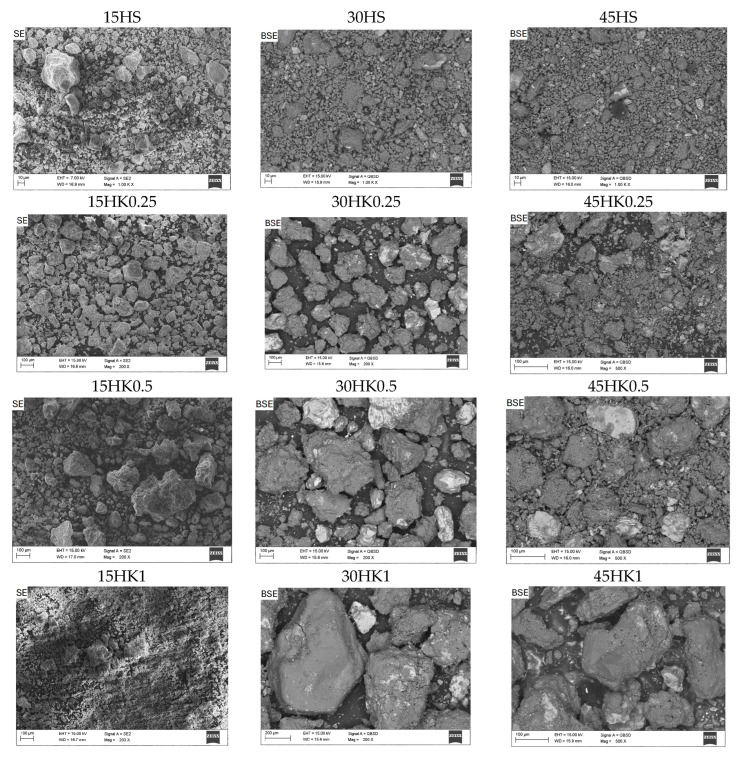
Surface structure of carbon-halloysite nanocomposites: HK025, HK05 and HK1 and HS after carbonization—mass concentration of sucrose: 15, 30 and 45 wt.% (SEM, SE, BSE detector, magnification 200–1000×).

**Figure 5:**
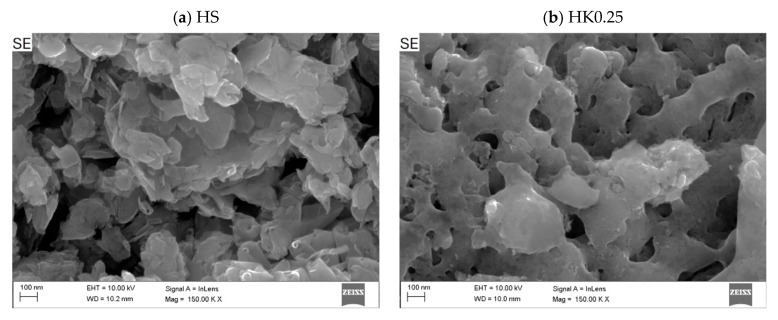

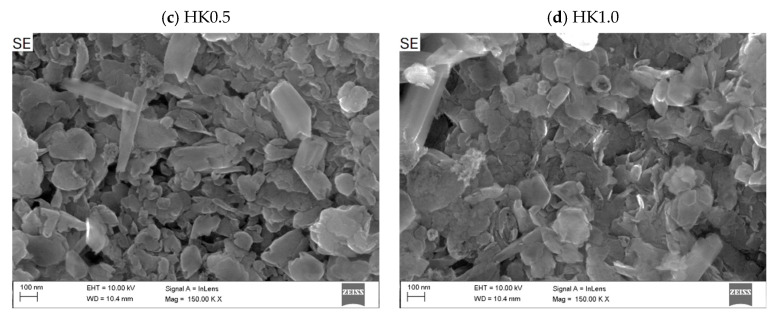
Surface structure of carbon-halloysite nanocomposites: HK025, HK05 and HK1 and HS after carbonization—mass concentration of sucrose: 45 wt.% (SEM, SE-In lens detector, magnification 150,000×).

**Figure 6:**
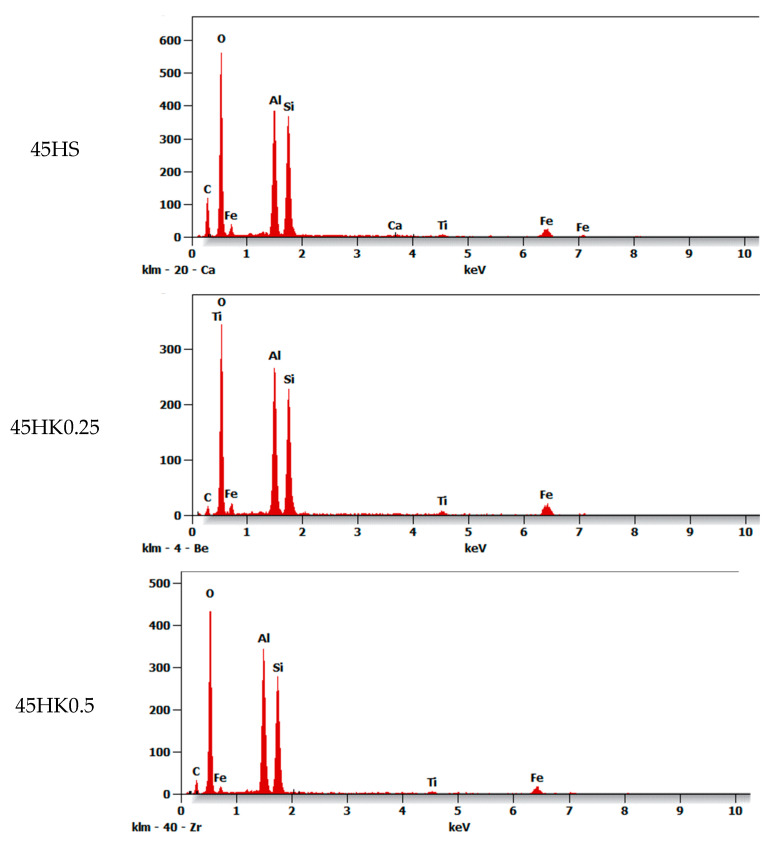

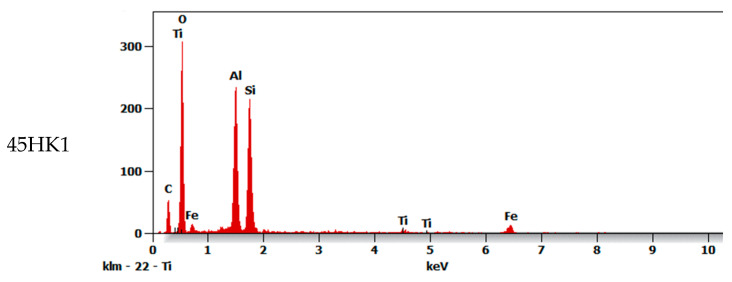
The EDS analysis of carbon-halloysite nanocomposite samples.

The SEM images of the halloysite and halloysite-based nanocomposite samples are presented in Figure 3, Figure 4 and Figure 5. The presence of deposited and distributed carbon in the obtained nanocomposites (Figure 4) is confirmed with the EDS analysis (Figure 6).

Based on the observation of the surface morphology of the HS and HK samples (Figure 3), it was found that as a result of the thermal calcination process, the surface of the particles exhibits characteristic changes associated with the mechanical effects of thermal stress relaxation and the formation of micro-cracks. As a result, this causes an increase in the surface roughness and morphological complexity of the surface. Observations of the surface of raw halloysite and synthesized composites using an analytical method based on chemical contrast (BSE, BackScattered Electron) detector confirmed that as a result of the carbonization process, carbon-halloysite nanocomposite materials were obtained. In their structure, the deposited carbon was identified analytically on the surfaces of halloysite particles (in SEM images these are particles of a darker color) (Figure 4). Few halloysite particles were also observed on which the entire available surface was not completely covered with a carbon layer (characteristic lighter areas). This indicates that the effects of the NPX separation process will be influenced by both sorption on the available surface developed in the structure of the deposited carbon layer (with a certain porosity, pore distribution and available adsorption surface, intrinsic chemical surface properties), as well as adsorption on the remaining surface of raw (or calcined) halloysite (competitive adsorption effects). Figure 5 shows the structure with visible micropores of carbon-halloysite nanocomposites marked HK025, HK05, and HK1, as well as HS after carbonization with a mass concentration of sucrose solution of 45 wt.% at a high magnification of 150,000×. The presence of carbon in the obtained nanocomposites is confirmed with the EDS analysis (Figure 6, Table 3). Based on the EDS chemical composition studies, it was found that with the increase in the mass concentration of sucrose in the solution (15 → 30 → 45 wt.%), the surface area covered with carbon in the halloysite particles increases (increased surface coverage efficiency), which is shown in Figure 6.

The comparison of the carbon content on the surface of raw halloysite (sample 45HS—12.2 wt.%) with calcined halloysite (samples: 45HK0.25—2.4 wt.%, 45HK0.5—11.3 wt.%, 45HK1—10.3 wt.%) indicates that the calcination process probably changes the morphology of the halloysite surface in such a way, that it makes it difficult to effectively deposit some carbon layer. In the case of calcined halloysite, the grain size affects the amount of carbon deposited—with the increase in grain size, its amount also increases.

### 3.2. FT-IR Analysis

**Figure 7:**
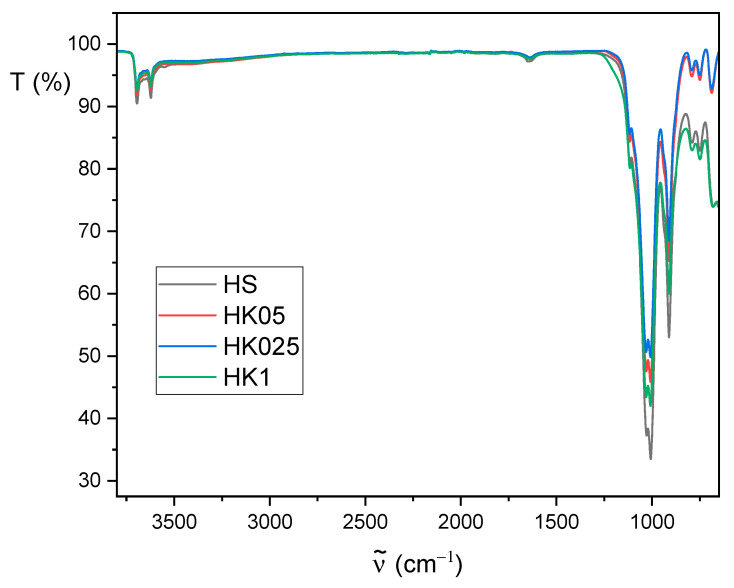
The FTIR spectra of HS, HK1, HK05 and HK025 samples.

The ATR Fourier-transform infrared spectroscopy (FT-IR) spectra of raw halloysite (HS) and calcined halloysite (HK) are presented in Figure 7. These spectra show characteristic bands for the kaolin-group minerals [60,61,62]: in the 3700–3600 cm^−1^ region, the vibration of the OH- group, and in the 1750–650 cm^−1^ region, bands assigned to apical Si–O (1107 cm^−1^) and to perpendicular stretching vibrations of Si–O–Si (1030 and 691 cm^−1^).

**Figure 8:**
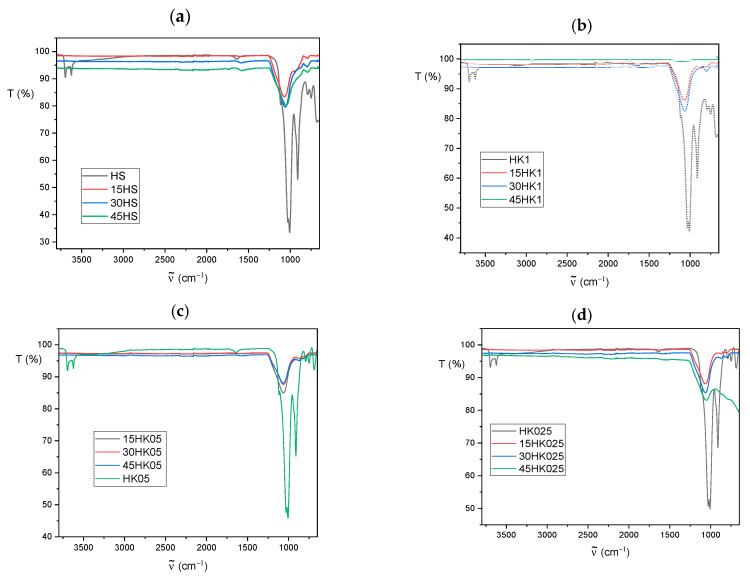
The FTIR spectra of: (**a**)—HS, 15HS, 30HS and 45HS samples, (**b**)—HK1, 15HK1, 30HK1 and 45HK1 samples, (**c**)—HK05, 15HK05, 30HK05 and 45HK05 samples, (**d**)—HK025, 15HK025, 30HK025 and 45HK025 samples.

Calcination reduces the intensity of the bands in the 1250–650 cm^−1^ range for HK1, HK05, HK025 samples in comparison to raw halloysite HS. In the case of all carbon-halloysite nanocomposites (Figure 8), the bands in the 3700–3600 cm^−1^ range assigned to the vibrations of the OH- groups are not observed, and the intensity of the bands in the range of 1250–650 cm^−1^ decreases significantly and changes their shape with increase in concentration of sucrose precursor in aqueous solution. These differences suggest the modifications on the surface of halloysite caused by the presence of carbon, confirming independently the conclusions derived based on data in Table 2 (specific BET surface area and total pore volume).

### 3.3. Adsorption Experiments

**Figure 9:**
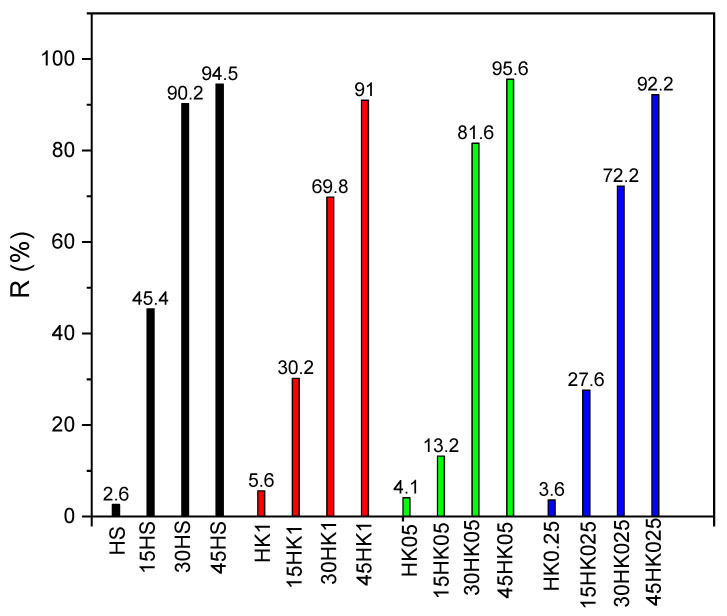
Dependence of the removal degree *R* (%) on the type of adsorbent for naproxen (*C*_0_ = 5 mg dm^−3^, adsorbent mass 0.1 g, interphase contact time 24 h).

The efficiencies of naproxen removal from aqueous solutions using raw and calcined halloysite, as well as various technical variants of carbon-halloysite nanocomposites as adsorbents are demonstrated in Figure 9. The results showed that the nanocomposites obtained using 45 wt.% sucrose solution as a carbon precursor (45HK05, *R* = 95.6%) and 45 HS, *R* = 94.5%) turned out to be comparable, the most effective adsorbents of naproxen from aqueous solutions. Considering these observations, the 45HS adsorbent with comparable adsorption abilities—however, without the need of prior calcination pretreatment—was selected for further research.

Based on the data presented in Figure 9, a clear increase in the removal degree *R* of the nanocomposite systems based on halloysite and carbon in removing NPX from aqueous solutions can be observed. Both in the case of HS and HK (in different variants), a significant increase in the NPX removal capacity was obtained. For the 45HS adsorbent, this is over 36-fold increase with respect to HS, for 45HK1—over 16-fold (with respect to HK1), for 45HK05—over 23-fold with respect to HK05, and for 45HK025—about 26-fold in comparison with the separation capacity of the HK025 adsorbent. The highest efficiency *R* (91–95.6%) was obtained for the highest concentrations of the carbon precursor (sucrose) in aqueous solutions, amounting to 45 wt.%. Compared to the basic adsorbents (HS, HK), acting as a carrier for deposited carbon (*R* 2.6–5.6%), these values are considerably much higher, enabling the practical use of this type of adsorption systems in advanced technological applications of wastewater treatment plants for the separation of pharmaceutical residues from various aqueous solutions.

In the case of the HS group adsorbents, the greatest efficiency increases are observed in the HS → 15HS → 30HS series, while the relative increase between the 30HS → 45HS adsorbents is only about 5%. A relatively systematic, gradual increase in separation efficiency was observed in the series: HK1 → 15HK1 → 30HK1 → 45HK1 and in the HK025 → 15HK025 → 30HK025 →45HK025 series. On the other hand, in the case of the HK05 group of nanocomposite adsorbents, a sudden, abrupt increase in separation efficiency was noted between the 15HK05 → 30HK05 variants.

The authors’ previous work on the separation capabilities of carbon-halloysite nanocomposites focused on corrugated cardboard as a carbon precursor [63] (for chloroxylenol and chlorophene), as well as on saccharose as the carbon precursor (adsorption of ketoprofen, naproxen and diclofenac—representing the non-steroidal anti-inflammatory drugs, NSAIDs [64], as well as paracetamol [65]).

In the case of corrugated cardboard as a carbon precursor, the maximum adsorption capacity, calculated on the basis of the Langmuir isotherm model was 14.48 mg g^−1^ for chloroxylenol, while for chlorophene it reached a value of 71.43 mg g^−1^. Analyzing the data from the point of view of process efficiency, for chloroxylenol the removal efficiency values were observed in the range of 67.3–97.7% (for halloysite alone—31.9%). For chlorophene, these were values in the range of 74.9–91.2% (where halloysite alone provided 24.6% only). For comparison, the commercial activated carbon AG-5 adsorbent enabled the achievement of separation efficiency of pharmaceuticals at the level of 72.7% for chloroxylenol, and 88.1% for chlorophene. The structure of carbonized cardboard alone allowed to obtain the removal efficiency of 87.1% for chlorophene and 94.7% for chloroxylenol. These values were slightly lower than the maximum values observed in the case of using the composite structure giving the highest results (91.2% for chlorophene, 97.7% for chloroxylenol). This indicates a clear, beneficial effect of the halloysite carrier enabling the increase in the active surface for the carbon layer, and thus a beneficial increase in the separation capacity [63].

The increase in separation efficiency of all three non-steroidal anti-inflammatory drugs, NSAIDs, tested in another authors work [64] was also clearly related to the increase in the concentration of the carbon layer deposited on the halloysite surface [64]. Depending on the carbon concentration in the nanocomposite structure (identified to be 2.2–6.7 wt.%), in the case of diclofenac, the removal efficiency values obtained ranged from 49 up to 72% (for the halloysite adsorbent alone—19% only), for ketoprofen from 60 to 90% (halloysite alone—16%) and for naproxen, these varied from 51 up to 85% (for halloysite alone—20%).

Also, depending on the concentration of the surface carbon layer (2.2–6.7 wt.%), the efficiency of paracetamol separation ranged from 42% (2.2 wt.% C) up to 93% (6.7 wt.% C). In the case of using halloysite alone as an adsorbent, the separation efficiency obtained was only 30% [65].

### 3.4. Effect of Adsorbent Dose

**Figure 10:**
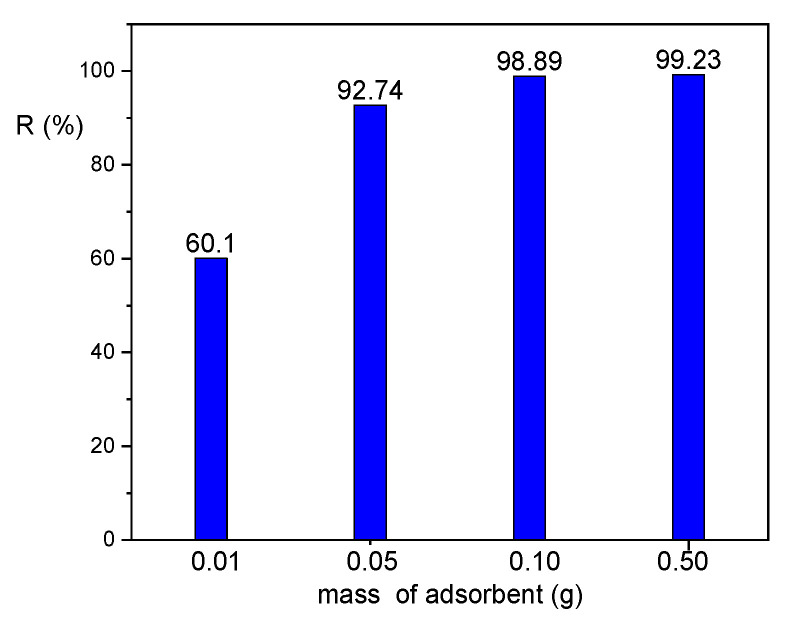
Dependence of the removal degree *R* (%) on the mass of the 45HS adsorbent for naproxen separation (*C*_0_ 5 mg dm^−3^, interphase contact time 24 h).

The influence of 45HS adsorbent dose on the adsorption of naproxen from aqueous solutions was studied. Aqueous solutions of naproxen of concentration 5 mg dm^−3^ and an adsorbent doses of: 0.10, 0.05 and 0.02 g (per 100 cm^3^) were applied for naproxen adsorption tests (interphase contact time 24 h). The results are presented in Figure 10. Removal efficiency of naproxen was the highest for the highest mass of adsorbent dosed, reaching values above 98% for its mass of 0.10 g per 100 cm^3^ (thus 1 g dm^−3^).

### 3.5. Effect of pH

**Figure 11:**
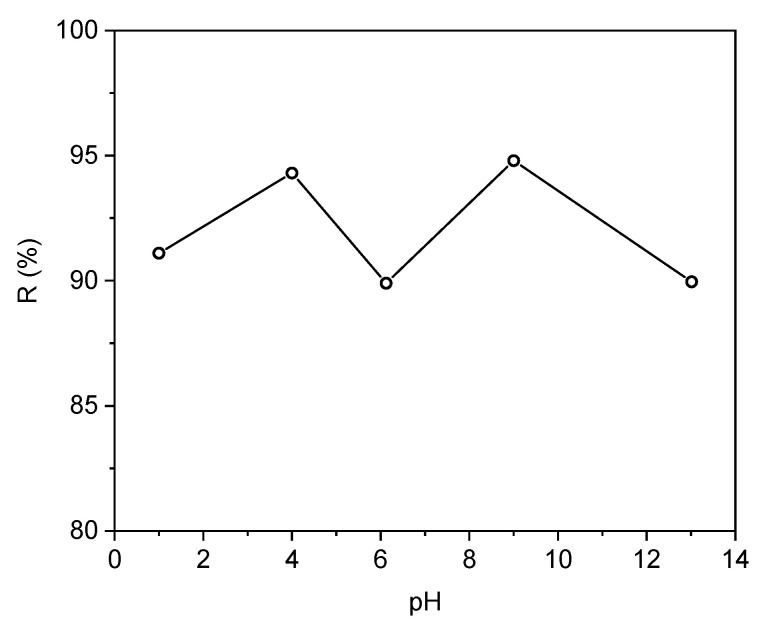
Dependence of solution’s pH on the naproxen separation degree *R* (%) for 45HS sample (*C*_0_ 5 mg dm^−3^, interphase contact time 24 h).

The adsorption of naproxen from its aqueous solution on carbonaceous adsorbents investigated in this work was mainly dependent on the physicochemical interactions between the functional groups within the complex naproxen molecule structure and the surface functional groups on the adsorbent [66]. Naproxen, with chemical characteristics of a weak organic acid, in aqueous solution coexists in both ionized and non-ionized forms. Proportion between both these forms depends on the solution’s pH. Net effect of ionized and non-ionized naproxen interactions with adsorbent surface may favor (or not) its adsorption on the carbon-halloysite composites. The effect of the solution’s pH on removal efficiency of naproxen using the 45HS adsorbent is presented in Figure 11.

### 3.6. Kinetic Models

The pseudo-first-order, pseudo-second-order kinetic models [67,68], and intraparticle diffusion model [69] were used for the modeling of adsorption of naproxen on the 45HS nanocomposite adsorbent sample. The use of kinetic models enabled the interpretation of the obtained measurement data and the identification of potential mechanisms and their stages influencing the process effects.

The pseudo-first-order and pseudo-second-order rate constants, *k*_1_ and *k*_2_, and the corresponding correlation coefficients (*R*^2^) are given in Table 4. Correlation coefficients clearly indicate the best possible fit of experimental data to the pseudo-second-order kinetic model, suggesting the electrochemical interactions between adsorbate and adsorbent surface. Moreover, data from the literature [70] indicate that the pseudo-second-order kinetic model shows a better fit to the experimental data when the adsorbent material is abundant with active sites, which results, among others, from the modification of the basic structure of the original adsorbent (e.g., modified clay [71], modified chitosan [72], surfactant-modified montmorillonite [70], modified hydrochar [73], modified bentonite [74], modified hydrogel [75]). The kinetic mechanism of the process is dominated in such cases by adsorption on the active sites of the adsorption surface, the number of which is significantly increased as a result of the structure modification preprocessing performed.

**Figure 12:**
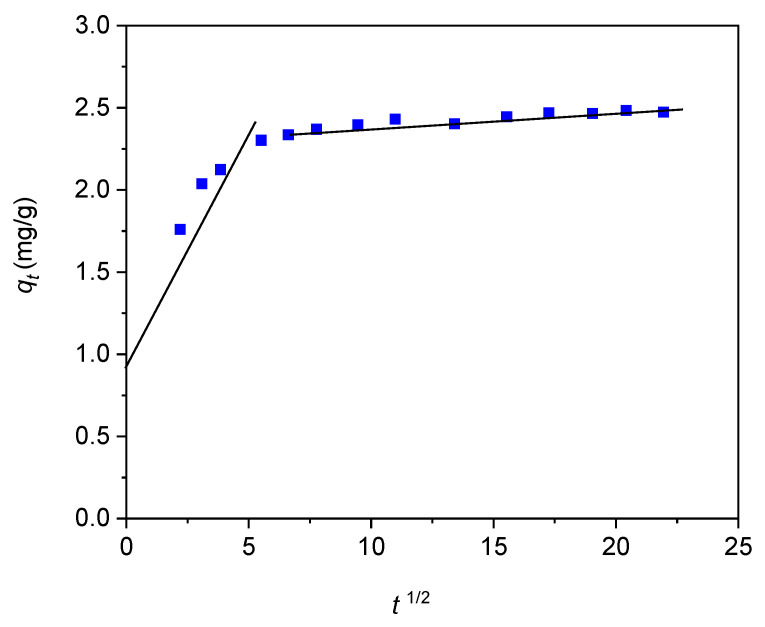
The intraparticle diffusion stages of naproxen on nanocomposite adsorbent 45HS.

The Weber–Morris internal diffusion model [69] was used to investigate the adsorption mechanism of naproxen from aqueous solutions on the 45HS adsorbent. The values of *k*_d1_, *k*_d2_ and *c*_1_, *c*_2_ determined from the slopes and intercepts of the first and second linear segment of graph presented in Figure 12 were collected in Table 5. The dependency of *q*_t_ vs. *t*^1/2^ as a multi-linear segment plot shows that at least two distinct steps are involved in the naproxen adsorption process. The first, faster step was attributed to the diffusion of adsorbate molecules to adsorbent outer surface, while the second one corresponds to slower stage, where intra-particle diffusion practically affects the overall adsorption process rate [70,76].

Two distinct sections of the Weber–Morris model dependence indicate a change in the kinetic mechanism of the process during its course. The first section does not pass through the point (0,0), which indicates that the entire adsorption is more complex in this case and is controlled by multiple sub-processes (segments in Figure 12). Increasing values of the model constant *c* for its subsequent stages indicate an growth in the diffusion resistance of the process in its subsequent stage.

### 3.7. Adsorption Isotherms

Fitting experimental adsorption equilibrium data to the most commonly used adsorption isotherm models was carried out using nonlinear regression (the Levenberg–Marquardt least squares method with the OriginLab software—Origin 2021b by OriginLab software). The results did not completely correlate with the Temkin, Dubinin–Radushkevich, Sips, and Freundlich models. For these adsorption isotherm models, the following values of the correlation coefficient *R*^2^ were obtained: 0.1009, 0.4138, 0.7607, and 0.8909, respectively.

**Figure 13:**
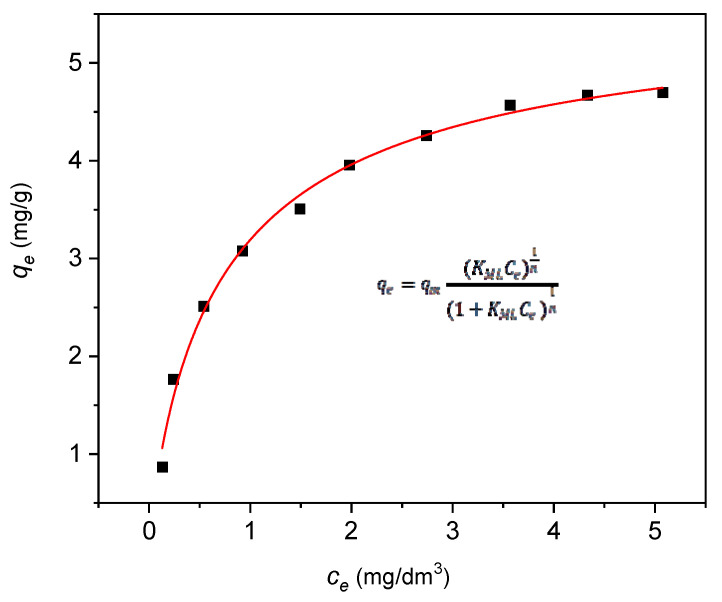
Experimental data of adsorption equilibrium for naproxen on adsorbent 45HS. The line represents the curve obtained by the application of the Langmuir multi-center adsorption isotherm model (4).

The best correlation of experimental data for naproxen and 45HS nanocomposite adsorbent was obtained for the Langmuir multi-center adsorption isotherm model Equation (4) (Figure 13). Equation parameters and correlation coefficient *R*^2^ were collected in Table 6:

(4) $$q_{e}=q_{m}\frac{{\left(K_{ML}C_{e}\right)}^{\frac{1}{n}}}{{\left(1+K_{ML}C_{e}\right)}^{\frac{1}{n}}}$$

where *q_e_*—the mass of a solute (adsorbate) adsorbed per 1 g of adsorbent at equilibrium (mg·g^−1^); *K_ML_*—Langmuir multi-center isotherm constant (dm^3^·mg^−1^)^1/*n*^; *q_m_*—maximum monolayer coverage capacity (mg·g^−1^); *n*—adsorption model index; *C_e_*—equilibrium concentration of a solute in the solution (mg·dm^−3^) [65].

The best model identified indicates a mechanism of multiple active centers on adsorbent surface without dissociation of naproxen particles.

The value of parameter *n* is fractional (Table 6), which indicates the adsorption mechanism of naproxen with a different number of adsorptive centers on the adsorbent surface. Therefore, more than one adsorbate molecule can occupy single adsorption center on the adsorbent surface.

The maximum adsorption capacity of the carbon-halloysite nanocomposite material for naproxen was 5.69 mg∙g^−1^ (Table 6). Kinetic measurements have shown that in practice this value is obtained after 100 min of interfacial contact. Data presented in the available literature on naproxen adsorption for alternative adsorbents indicate that the efficiency of the obtained adsorption surface is higher than the separation capabilities of molecularly imprinted polymers (1.93 mg∙g^−1^), montmorillonite (1.96 mg∙g^−1^) and chitosan-modified waste tire rubber (2.30 mg∙g^−1^) [46,77,78,79]. It also significantly exceeds the value for polymeric resins (0.83 mg∙g^−1^) [80], but is comparable with halloysite functionalized with PAMAM dendrimer (5.96 mg∙g^−1^) [77]. More complex adsorbent configurations presented in the literature, such as: nanographene (17.80 mg∙g^−1^) [81], β-cyclodextrin-PVP-clay (30.64 mg∙g^−1^) [82], UiO-66/UiO-66-NH_2_ (90 mg∙g^−1^) [83] enable obtaining higher unit adsorption capacities in relation to naproxen. However, these represent systems with rather complex structure, with relatively high production costs, which effectively eliminates their potential common use in industrial-scale environmental protection. It should be emphasized that the aim of the research conducted by the authors in this study was to develop a formula and methodology for obtaining the most effective and technologically simple adsorbent, which, due to the possible lowest manufacturing costs, can be widely used in sewage treatment systems.

## 4. Conclusions

Based on the results of the research and their technological interpretation, the following conclusions can be formulated:

(1) The deposition of a carbon layer onto the halloysite carrier, represented by both raw halloysite (HS) and halloysite subjected to a controlled calcination process (HK), enabled the practical removal of the previous technological barrier related to the very low adsorptive separation efficiency of natural clays in relation to NPX with its characteristic chemical structure and charge distribution properties.
(2) The reduction in the specific surface area of HS- and HK-based adsorbents, related to the surface deposition of carbon and the resulting changes in such obtained surface morphology of nanocomposites, was overcompensated by the much higher affinity of surface functional groups for NPX, which is directly related to the increased technological effect of NPX separation.
(3) The SEM/EDS analysis indicates a combined effect of the adsorption process on and within the carbon layer and on the remaining unoccupied internal and external surface of the halloysite carrier (competitive adsorption on the actual surface structure).
(4) The kinetic conditions of the process (pseudo-second-order kinetic model) indicate a favorable increase in the number of active sites formed after the deposition of a carbon layer on the surface of halloysite support. The presented kinetic data of the adsorption process and their theoretical interpretation refer to the aqueous model solution of naproxen.
(5) The validation of the Langmuir multi-center adsorption isotherm model indicates a separation mechanism associated with the occurrence of multiple active centers on the nanocomposite adsorbent surface and the separation effect without dissociation of naproxen particles. The presented equilibrium data of the adsorption process and their theoretical interpretation refer to the aqueous model solution of naproxen.
(6) The proposed nanocomposite preparation method is cost-effective, technologically simple, and environmentally friendly, utilizing sucrose, water, and naturally occurring halloysite. Given the substantial NPX removal efficiency demonstrated in this study, the method holds promise for widespread application in the economical and effective removal of pharmaceutical contaminants from aqueous systems.

## Figures and Tables

**Table 1:** Chemical structure and selected properties of naproxen.

Compound	Naproxen
Molecular structure	** 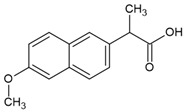 **
IUPAC name	(2S)-2-(6-methoxynaphthalen-2-yl)propanoic acid
Chemical formula	C_14_H_14_O_3_
Molecular weight	230.26 g mol^−1^
Water solubility	15.9 mg·dm^−3^ (25 °C)
pK_a_ *	4.15
logK_OW_ **	3.18

* pK_a_ = −logK_a_ (dissociation constant, 20 °C), ** K_OW_—octanol–water distribution coefficient.

**Table 2:** Structural parameters of carbon-halloysite nanocomposites tested.

Adsorbent	*S*_BET_ (m^2^ g^−1^)	*V*_t_ (cm^3^ g^−1^)	*V*_mi_ (cm^3^ g^−1^)	*V*_me_ (cm^3^ g^−1^)	Mesoporosity (%)
HS	45.64	0.1925	0.0036	0.1906	99
15HS	27.23	0.1348	0.0027	0.1321	98
30HS	43.37	0.1559	0.0015	0.1544	99
45HS	54.75	0.1340	0.0057	0.1283	96
HK1	62.58	0.1896	0.0020	0.1876	99
15HK1	29.67	0.1423	0.0016	0.1407	99
30HK1	31.20	0.1369	0.0037	0.1332	97
45HK1	41.87	0.1508	0.0039	0.1469	97
HK05	69.74	0.1891	0.0020	0.1871	99
15HK05	33.73	0.1611	0.0009	0.1602	99
30HK05	39.67	0.1695	0.0031	0.1664	98
45HK05	42.15	0.1671	0.0022	0.1649	99
HK025	64.01	0.1757	0.0018	0.1739	97
15HK025	30.35	0.1410	0.0007	0.1403	99
30HK025	34.61	0.1518	0.0013	0.1505	99
45HK025	42.15	0.1671	0.0022	0.1649	99

**Table 3:** Chemical elementary composition (EDS) of the selected surface areas of nanocomposite samples (wt. %).

Composite	C	O	Al	Si	Ti	Fe
45HS	12.2	42.3	15.0	17.1	1.3	11.7
45HK0.25	2.4	42.4	18.7	19.6	2.2	14.7
45HK0.5	11.3	41.4	16.0	16.1	1.3	13.2
45HK1	10.3	42.6	16.5	18.6	1.2	11.0

**Table 4:** Kinetic parameter values corresponding to naproxen adsorption on 45HS nanocomposite.

Adsorbate	Pseudo-First-Order Kinetic Model		Pseudo-Second-Order Kinetic Model	
Naproxen	*k*_1_ (min^−1^)	*R* ^2^	*k*_2_ (g mg^−1^ min^−1^)	*R* ^2^
	0.00923	0.85353	0.03166	0.9991

**Table 5:** The Weber–Morris intraparticle diffusion model parameter values.

Adsorbate	*k*_d1_ (mg g^−1^ min^−1/2^)	*c*_1_ (mg g^−1^)	*R*_1_^2^	*k*_d2_ (mg g^−1^ min^−1/2^)	*c*_2_ (mg g^−1^)	*R*_2_^2^
Naproxen	0.4101	0.938	0.6523	0.0096	2.372	0.7456

**Table 6:** Langmuir multi-center adsorption isotherm parameters, and correlation coefficient *R*^2^ for the adsorption of naproxen on 45HS nanocomposite adsorbent.

Langmuir Multi-Center Model			
*K_MF_* (dm^3^∙mg^−1^)^1/n^	*q_m_* (mg∙g^−1^)	*n*	*R* ^2^
1.34	5.69	1.19	0.9934

## Data Availability

The original contributions presented in this study are included in the article. Further inquiries can be directed to the corresponding authors.
